# IP3R1 regulates Ca^2+^ transport and pyroptosis through the NLRP3/Caspase-1 pathway in myocardial ischemia/reperfusion injury

**DOI:** 10.1038/s41420-021-00404-4

**Published:** 2021-02-10

**Authors:** Guixi Mo, Xin Liu, Yiyue Zhong, Jian Mo, Zhiyi Li, Daheng Li, Liangqing Zhang, Yijun Liu

**Affiliations:** Department of Anesthesiology, Affiliated Hospital of Guangdong Medical university, Zhanjiang, Guangdong P.R. China

**Keywords:** Calcium channels, Thromboembolism

## Abstract

Intracellular ion channel inositol 1,4,5-triphosphate receptor (IP3R1) releases Ca^2+^ from endoplasmic reticulum. The disturbance of IP3R1 is related to several neurodegenerative diseases. This study investigated the mechanism of IP3R1 in myocardial ischemia/reperfusion (MI/R). After MI/R modeling, IP3R1 expression was silenced in myocardium of MI/R rats to explore its role in the concentration of myocardial enzymes, infarct area, Ca^2+^ level, NLRP3/Caspase-1, and pyroptosis markers and inflammatory factors. The adult rat cardiomyocytes were isolated and cultured to establish hypoxia/reperfusion (H/R) cell model. The expression of IP3R1 was downregulated or ERP44 was overexpressed in H/R-induced cells. Nifedipine D6 was added to H/R-induced cells to block Ca^2+^ channel or Nigericin was added to activate NLRP3. IP3R1 was highly expressed in myocardium of MI/R rats, and silencing IP3R1 alleviated MI/R injury, reduced Ca^2+^ overload, inflammation and pyroptosis in MI/R rats, and H/R-induced cells. The binding of ERP44 to IP3R1 inhibited Ca^2+^ overload, alleviated cardiomyocyte inflammation, and pyroptosis. The increase of intracellular Ca^2+^ level caused H/R-induced cardiomyocyte pyroptosis through the NLRP3/Caspase-1 pathway. Activation of NLRP3 pathway reversed the protection of IP3R1 inhibition/ERP44 overexpression/Nifedipine D6 on H/R-induced cells. Overall, ERP44 binding to IP3R1 inhibits Ca^2+^ overload, thus alleviating pyroptosis and MI/R injury.

## Introduction

It is widely accepted that ischemic disease represents a major health threat as it leads to numerous mortality every year across the world^1^. Ischemia/reperfusion (I/R) injury, as a complicated pathological process, includes nonspecific damage caused by ischemia and injury induced by reactive oxygen, nitrogen species, and inflammatory cytokines during reperfusion^2^. Myocardial I/R (MI/R) injury induces myocardial cell apoptosis, and contributes to adverse cardiovascular outcomes, heart dysfunction, and structural damage^3^. Intracellular Ca^2+^ ([Ca^2+^]i) overload is a key cause of cellular injury and myocardial apoptosis^4^. Strong activation of Ca^2+^ channels may be an essential mechanism for neuronal death after cerebral I/R injury^5^. Cardiomyocyte cell death in response to I/R results from the increases in Ca^2+^ ^6^. I/R injury activates endoplasmic reticulum (ER) stress in cardiomyocytes, and persistent ER stress with an increase in [Ca^2+^]i concentration from the ER through the inositol 1,4,5 trisphosphate receptor (IP3R), leads to cardiomyocyte apoptosis^7^. These references provide a novel perspective for I/R treatment that degrading [Ca^2+^]i concentration in view of ER stress and IP3R may be beneficial to relieve MI/R injury.

Elevated levels of IP3 in adult cardiomyocytes are associated with the development of cardiac hypertrophy, arrhythmias, and heart failure^8^. Increased expression of IP3R1 is also observed after hypoxia/reperfusion (H/R) injury of spinal cord dorsal column in vitro^9^. IP3R, a [Ca^2+^]i release channel is predominately expressed on the ER membranes^10^. Endoplasmic reticulum resident protein 44 (ERP44) binds to IP3R1 and regulates [Ca^2+^]i concentrations by modulating IP3R1 activity^11^. ERP4 has also been shown to participate in Ca^2+^ homeostasis, maturation of secretory proteins within ER, cardiac development, control of blood pressure, and ER stress activation^12,13^. But little is known about the role of ERP44 in MI/R injury.

Pyroptosis is a type of inflammatory programmed cell death induced by pro-inflammatory mediators and exhibits morphological characteristics that are different from apoptosis and necrosis^14–16^. Pyroptosis is characterized by activation of nod-like receptor protein-3 (NLRP3) inflammasome and Caspase and the release of interleukin (IL)-1β and IL-18^17,18^. The role of pyroptosis in cancers, cardiovascular diseases, and microbial infection-related diseases is a hot spot in recent years^17,19,20^. As has been verified, cell pyroptosis is also closely involved in MI/R injury^21^. Inhibited pyroptosis helps to attenuate renal I/R injury^22^. Importantly, Ca^2+^ signaling is critical for NLRP3 inflammasome activation to induce pyroptosis^23^. However, the role of IP3R/ERP44 in pyroptosis in MI/R injury remains unknown.

Given the former analyses, whether ERP44 binding to IP3R can regulate Ca^2+^ overload and pyroptosis through the NLRP3/Caspase-1 pathway and thus plays a protective role in MI/R injury has not been reported at home and abroad. Therefore, the aim of this study is to study the protective mechanism of ERP44 binding to IP3R against MI/R injury and to provide a new theoretical basis for the management of MI/R injury.

## Results

### Inhibition of IP3R1 reduces MI/R-induced pyroptosis and alleviates myocardial injury

**Fig. 1:**
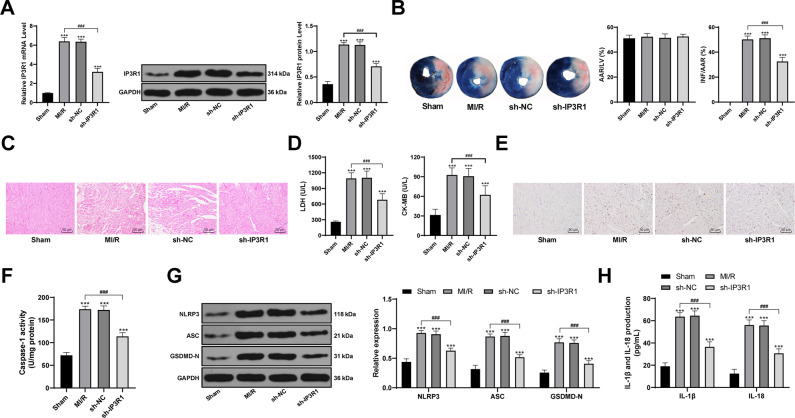
Inhibition of IP3R1 reduces MI/R-induced pyroptosis and alleviates myocardial injury. **A** RT-qPCR and western blot were used to detect the levels of IP3R1 in the myocardium of rats after MI/R and shRNA intervention; **B** TTC staining was used to detect the infarct size of MI/R rats. AAR/LV reflects the degree of ischemia, INF/AAR reflects the degree of infarction, *n* = 3; **C** HE staining was used to observe the pathological changes of myocardium of MI/R rats, *n* = 6. **D** the effect of IP3R1 on the concentration of myocardial enzymes in MI/R rats, *n* = 21; **E** immunohistochemical observation of GSDMD-positive expression, *n* = 6; **F** colorimetric method was used to detect the activity of Caspase-1, *n* = 6; **G** Western blot detected the levels of NLRP3, ASC, and GSDMD-N, *n* = 6; **H** ELISA was used to measure the levels of IL-1β and IL-18, *n* = 6; compared with the sham group, ***p < 0.001; for pairwise comparison, ^###^*p* < 0.001. Data in panels A/B/D/F were analyzed using one-way ANOVA and data in panels G/H were analyzed using two-way ANOVA. Tukey’s multiple comparison test was applied for the post hoc test after ANOVA.

We established the MI/R model in SD rats by surgery, and found that IP3R1 expression in cardiomyocytes of rats after I/R treatment was significantly increased (*p* < 0.05; Fig. 1A). Compared to those in MI/R rats, the INF/ARR in the heart of sh-IP3R1-treated rats was reduced, the cardiomyocytes were orderly arranged, and the concentration of myocardial enzymes was significantly decreased (all *p* < 0.05; Fig. 1B–D). Then we measured the inflammasome proteins and pyroptosis markers in cardiomyocytes of MI/R rats. In MI/R rats, the expression of NLRP3 and ASC was significantly increased, Caspase-1 was activated, GSDMD-positive rate in cardiomyocytes was elevated, and a large number of inflammatory factors (IL-1β and IL-18) were released. However, the levels of pyroptosis markers and the release of inflammatory factors were alleviated after inhibition of IP3R1 (all *p* < 0.05; Fig. 1E–H). It is suggested that inhibiting IP3R1 expression can reduce pyroptosis induced by MI/R and alleviate myocardial injury.

### Inhibition of IP3R1 attenuates H/R-induced pyroptosis and inflammation in cardiomyocytes

**Fig. 2:**
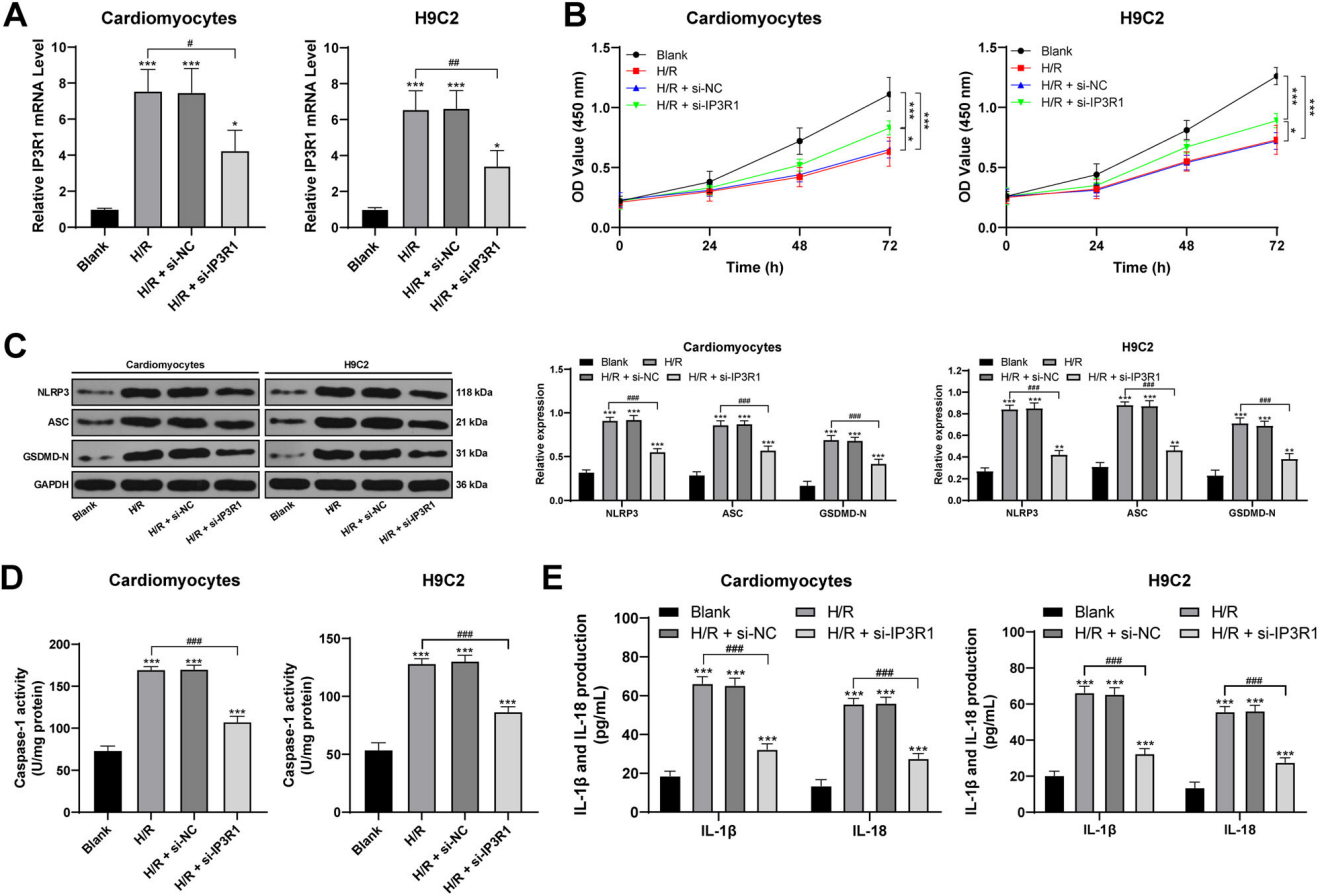
Inhibition of IP3R1 attenuates H/R-induced pyroptosis and inflammation in cardiomyocytes. **A** RT-qPCR was used to detect the efficiency of siRNA transfection; **B** CCK-8 was used to detect cell viability; **C** Western blot was used to detect the levels of pyroptosis-related proteins; **D** colorimetric method was used to detect the activity of Caspase-1; **E** ELISA was used to detect the levels of IL-1β and IL-18. Cardiomyocytes are isolated and cultured from adult rat myocardium. Compared to the blank group, *p < 0.05, **p < 0.01, ***p < 0.001; for pairwise comparison, ^#^*p* < 0.05, ^##^*p* < 0.01, ^###^*p* < 0.001. Data in panels **A**/**D** were analyzed using one-way ANOVA and data in panels **B**/**C**/**E** were analyzed using two-way ANOVA. Tukey’s multiple comparison test was applied for the post hoc test after ANOVA.

To further study the role of IP3R1 in MI/R, we purchased H9C2 cell line, and isolated and cultured adult rat cardiomyocytes, and treated these cells with H/R. After H/R treatment, the cell activity was decreased, the levels of intracellular pyroptosis markers were increased significantly (the expression of NLRP3 and ASC upregulated, GSDMD-N increased, Caspase-1 was activated), and the levels of inflammatory factors were significantly elevated (all *p* < 0.05; Fig. 2B–E). After inhibiting IP3R1 expression in cells, these trends were reversed. The results were consistent with the results in vivo, which revealed that IP3R1 regulated the level of intracellular pyroptosis to regulate MI/R injury.

### Upregulation of IP3R1 leads to intracellular Ca^2+^ overload in cardiomyocytes

**Fig. 3:**
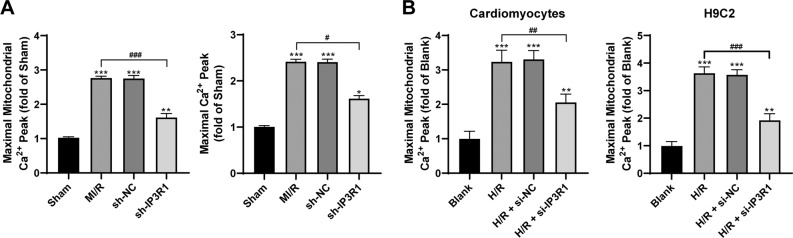
Upregulation of IP3R1 leads to intracellular Ca^2+^ overload in cardiomyocytes. **A**, **B** Fluo 4-AM and Rhod2 AM were used to observe the changes of Ca^2+^ levels in cells and mitochondria; *n* = 6. Compared with the sham group, *p < 0.05, **p < 0.01, ***p < 0.001; for pairwise comparison, ^#^*p* < 0.05, ^##^*p* < 0.01, ^###^*p* < 0.001. Data were analyzed using one-way ANOVA and Tukey’s multiple comparison test.

IP3R1 is a calcium transport related protein. To further study the mechanism of IP3R1 in MI/R, we detected the level of Ca^2+^ in mitochondria and cardiomyocytes of rats with MI/R and in H/R-challenged cells, and found that the level of Ca^2+^ was increased evidently (both *p* < 0.05; Fig. 3A). After downregulating the expression of IP3R1 in myocardial tissues of MI/R rats by shRNA, the Ca^2+^ level in mitochondria was significantly decreased (both *p* < 0.05; Fig. 3A); similar results were found in H/R-challenged cells (all *p* < 0.05; Fig. 3B). It is suggested that inhibiting IP3R1 expression can inhibit the intracellular Ca^2+^ overload induced by MI/R.

### ERP44 binding to IP3R1 inhibits mitochondrial Ca^2+^ overload in cardiomyocytes

**Fig. 4:**
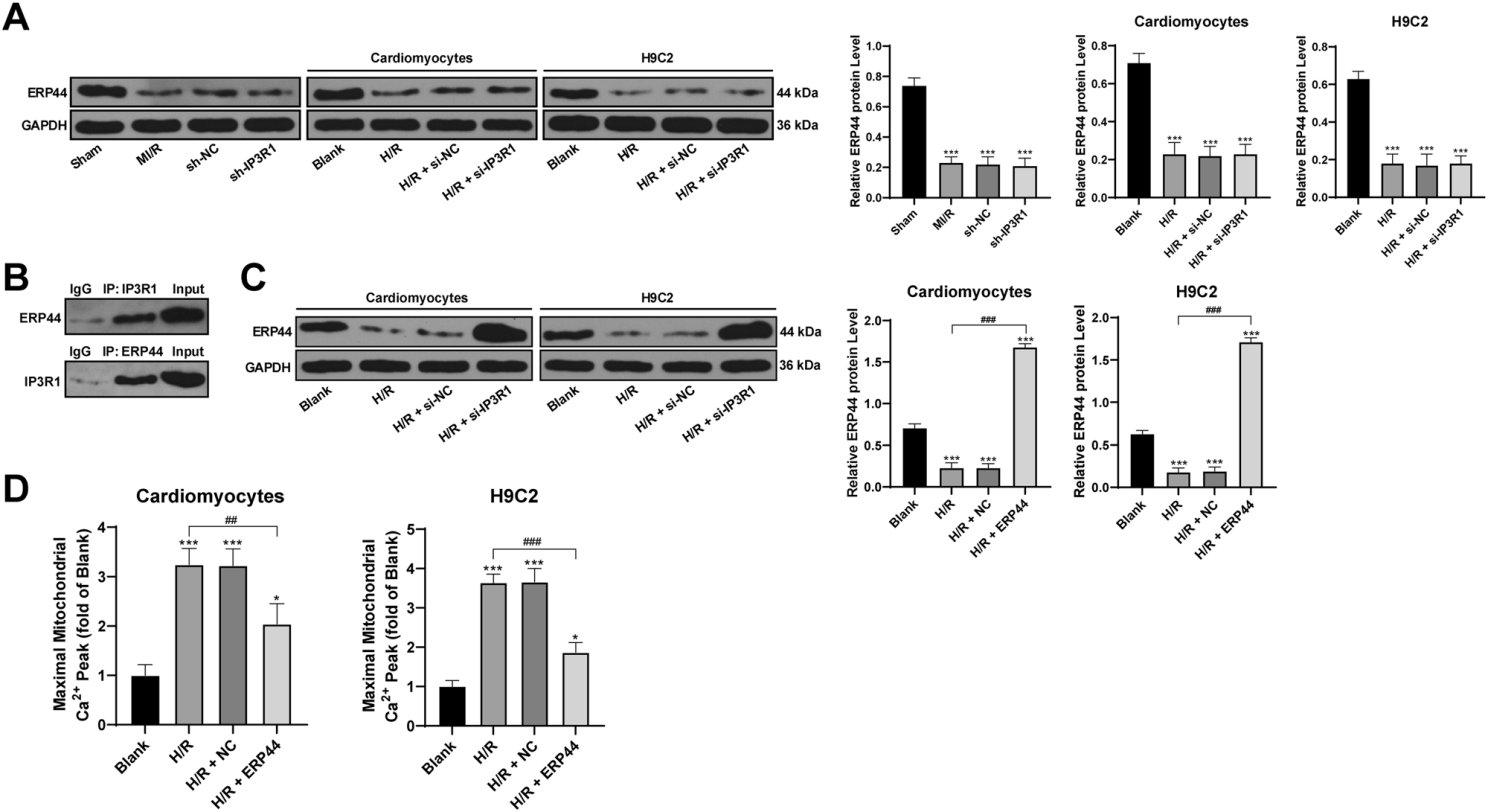
Binding of ERP44 to IP3R1 inhibits the I/R-induced mitochondrial Ca^2+^ overload. **A**, **C** Western blot was used to detect the level of ERP44 protein; **B** immunoprecipitation was used to detect the binding relationship between IP3R1 and ERP44; **D** Rhod2 AM fluorescent labeling was used to observe the changes of intracellular Ca^2+^ level. Compared to the blank group, *p < 0.05, ***p < 0.001; for pairwise comparison, ^##^*p* < 0.01, ^###^*p* < 0.001. All experiments were *p*erformed three times independently. Data were analyzed using one-way ANOVA and Tukey’s multiple comparison test.

IP3R1 regulates the direct calcium transfer from endoplasmic reticulum to mitochondria^24^, while ERP44 is important in the development of embryonic heart, and also in regulating the Ca^2+^ signal of cardiomyocytes^24^. ERP44 could directly inhibit the activity of IP3R1^25^. Thus, we examined ERP44 expression in the myocardium of MI/R rats and in the cells treated with H/R. After MI/R treatment and H/R treatment, the expression of ERP44 was significantly decreased (*p* < 0.05; Fig. 4A). In addition, immunoprecipitation showed that ERP44 could directly bind to IP3R1 (Fig. 4B). Subsequently, we overexpressed ERP44 in cells (*p* < 0.05; Fig. 4C), and found that the level of Ca^2+^ in H/R mitochondria was decreased clearly after overexpression of ERP44 (all *p* < 0.05; Fig. 4D), indicating that the binding of ERP44 to IP3R1 inhibited the I/R-induced mitochondrial Ca^2+^ overload.

### Overexpression of ERP44 attenuates H/R-induced cardiomyocyte pyroptosis

**Fig. 5:**
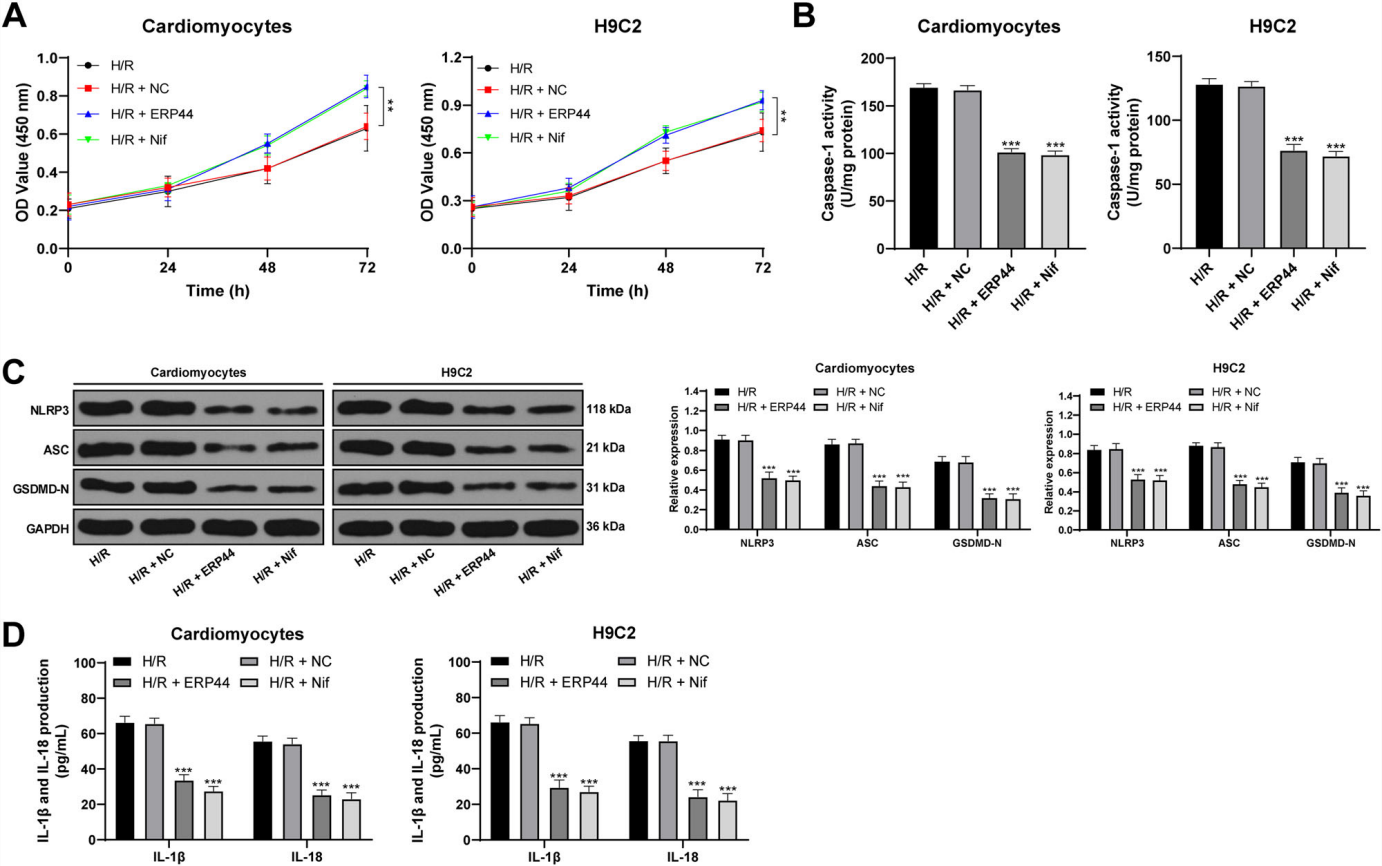
Overexpression of ERP44 attenuates H/R-induced cardiomyocyte pyroptosis. **A** CCK-8 was used to detect cell viability; **B** colorimetric assay was used to detect Caspase-1 activity; **C** western blot was used to detect the levels of pyroptosis proteins; **D** ELISA was used to detect the levels of IL-1β and IL-18. Nif represents calcium channel blocker Nifedipine D6. Compared to the H/R group, **p < 0.01, ***p < 0.001. All experiments were performed three times independently. Data in panel **B** were analyzed using one-way ANOVA and data in panels **A**/**C**/**D** were analyzed using two-way ANOVA. Tukey’s multiple comparison test was applied for the post hoc test after ANOVA.

To investigate whether the mitochondrial Ca^2+^ overload regulated by the combination of ERP44 and IP3R1 can modulate the pyroptosis of cardiomyocytes mediated through the NLRP3/Caspase-1 pathway, we overexpressed ERP44 in H/R-challenged cells. After ERP44 overexpression, the activity of H/R-challenged cells was increased, the levels of NLRP3 and ASC were decreased, the activated Caspase-1 was decreased, the GSDMD-N was reduced, and the levels of inflammatory factors (IL-1β and IL-18) were downregulated. This effect was similar to that of H/R cells treated with calcium channel blocker Nifedipine D6 (all *p* < 0.05; Fig. 5A–D), indicating that overexpression of ERP44 could inhibit the transport of Ca^2+^ by binding to IP3R1, thus reducing mitochondrial Ca^2+^ overload in H/R-induced cells and alleviating cardiomyocyte pyroptosis.

### Activation of NLRP3 inflammasome pathway reverses the protection of ERP 44 against H/R injury

**Fig. 6:**
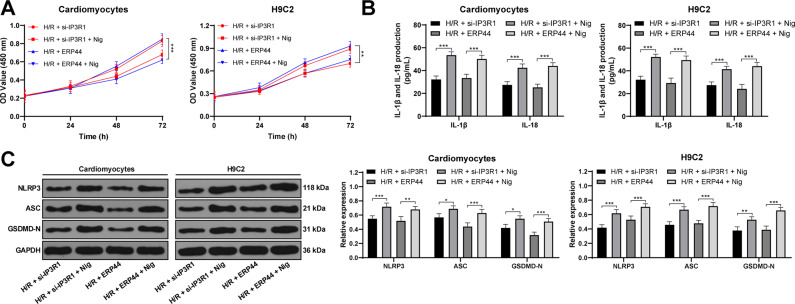
Activation of NLRP3 pathway reverses the protection of ERP 44 against H/R injury. **A** CCK-8 was used to detect cell viability; **B** ELISA was used to detect the levels of IL-1β and IL-18 in cells; **C** western blot was used to detect the levels of pyroptosis markers in cells. Nig represents an activator of NLRP3. *p < 0.05, **p < 0.01, ***p < 0.001. All experiments were performed three times independently. Data were analyzed using two-way ANOVA and Tukey’s multiple comparison test.

To verify that the intracellular mitochondrial Ca^2+^ overload regulated by the binding of ERP44 and IP3R1 is controlled by the pyroptosis mediated by the NLRP3/Caspase-1 pathway, we intervened the NLRP3/Caspase-1 pathway. Nigericin is an antibiotic derived from streptomyces hygroscopicus and can be used as an activator of NLRP3^26^. We added Nigericin to H/R cells overexpressing ERP44 or silencing IP3R1. Activation of NLRP3 reversed the protective effect of overexpression of ERP44 or inhibition of IP3R1 on H/R cells, as evidenced by a significant decrease in cell activity, an increase in pyroptosis markers, and an increase in the release of inflammatory factors (all *p* < 0.05; Fig. 6A–C). These results further confirmed that the increase of intracellular Ca^2+^ level regulated by ERP44 or IP3R1 can cause H/R-induced cardiomyocyte pyroptosis through the NLRP3/Caspase-1 pathway, thus regulating MI/R injury.

## Discussion

This study provided evidence that IP3R1 is highly expressed in myocardium of MI/R rats and IP3R1 promotes mitochondrial Ca^2+^ overload and pyroptosis in MI/R rats. Importantly, ERP44 could bind to IP3R1 to inhibits the transport of Ca^2+^, thus reducing [Ca^2+^]i and alleviating pyroptosis and MI/R injury through the NLRP3/Caspase-1 pathway.

IP3R1 expression is increased markedly in pigs exposed to high stress^27^. Significant increases in IP3R1 levels in renal tissues are observed in animal models of renal insufficiency^28^. We found that IP3R1 expression in cardiomyocytes of rats after MI/R was remarkably increased. IP3R1 expression is increased in H/R injury of spinal cord white matter^9^. Recent studies have shown that cell pyroptosis is related to I/R injury^22,29^. Pyroptosis is defined as a Caspase-1-dependent mechanism, and NLRP3 is indispensable for the activation of Caspase-1^30,31^. The pyroptosis (the expression of NLRP3 and ASC was decreased, Caspase-1 was inactivated, GSDMD-positive rate) in cardiomyocytes of MI/R rats was inhibited and the release of inflammatory factors (IL-1β and IL-18) were alleviated after inhibition of IP3R1. NLRP3 inflammasomes consist of NLRP3, ASC, and procaspase-1^32^. It is well accepted that Caspase-1 and GSDMD are pyroptosis-related proteins^33^ and GSDMD controls the release of IL-1β and IL-18^34^. Elevations IL-1β and IL-18 contribute to cell pyroptosis^35^. Inhibiting NLRP3-mediated pyroptosis is neuroprotective against cerebral I/R injury^36^. Taken together, inhibiting IP3R1 expression can reduce the levels of pyroptosis markers induced by MI/R and alleviate myocardial injury. When IP3R1 expression was decreased using siRNA, the activity of H9C2 cells was increased and the levels of pyroptosis markers and inflammatory proteins were decreased. The results were consistent with the results in vivo, suggesting that IP3R1 downregulation inhibited intracellular pyroptosis to alleviate the H/R injury.

Increased activity of IP3Rs causes mitochondrial Ca^2+^ overload which can lead to apoptosis^9^. During the process of MI/R injury, the [Ca^2+^]i concentration continues to increase and leads to cardiomyocyte apoptosis and myocardial damage^37^. After downregulating IP3R1 expression in myocardial tissue of MI/R rats using shRNA, the Ca^2+^ level in mitochondria was clearly decreased; similar results were found in H/R-challenged cells. Sustained global elevation of [Ca^2+^]i eventually results in zymogen activation and inflammatory injury^38^. After silencing IP3R1, the inflow of Ca^2+^ and the release of [Ca^2+^]i stores are reduced in cells with TNF-α-induced [Ca^2+^]i overload^39^. To reduce [Ca^2+^]i and maintain Ca^2+^ homeostasis can protect cardiomyocytes from I/R injury^4^. These results suggested that inhibiting IP3R1 expression can inhibit the [Ca^2+^]i overload induced by MI/R. Then we investigated how did IP3R1 inhibition inhibit the [Ca^2+^]i overload. Besides, ERP44 could directly inhibit the activity of IP3R1^25^ and regulate the Ca^2+^ signal of cardiomyocytes^24^. Therefore, we detected ERP44 expression in the myocardium of MI/R rats and in the cells treated with H/R. After MI/R treatment and H/R treatment, the expression of ERP44 was notably decreased. In addition, immunoprecipitation showed that ERP44 could directly bind to IP3R1. ERP44 directly interacts with IP3R1 and inhibits IP3-induced Ca^2+^ release^13,40^. IP3-induced Ca^2+^ release is decreased in HeLa cells overexpressing ERP44 through IP3R channels^40^. The level of Ca^2+^ in H/R-induced mitochondria was decreased clearly after overexpression of ERP44, indicating that the binding of ERP44 to IP3R1 inhibited the MI/R-induced mitochondrial Ca^2+^ overload.

Perturbation of intracellular ion homeostasis is a key signal for activation of NLRP3 inflammasome and subsequent pyroptosis^41^. Ca^2+^ influx is required for NLRP3 activation and cytokine release^42^. Moreover, we evaluated whether ERP44/IP3R1-mediated [Ca^2+^]i level monitors the pyroptosis. After ERP44 overexpression, the activity of H/R-challenged cells was increased, the levels of pyroptotic markers (NLRP3, ASC, Caspase-1, and GSDMD-N) were reduced, and IL-1β and IL-18 were downregulated. Inhibition of IP3R channel has been reported to attenuate Ca^2+^ mobilization and NLRP3 inflammasome activation^23,43^. This effect of ERP44 overexpression was similar to that of H/R cells treated with calcium channel blocker Nifedipine D6. Blocking Ca^2+^ mobilization inhibits activation of the NLRP3 inflammasome complex^23^. In brief, overexpression of ERP44 could inhibit the transport of Ca^2+^ by binding to IP3R1, thus reducing mitochondrial Ca^2+^ overload in H/R cells and alleviating cardiomyocyte pyroptosis. To verify that the [Ca^2+^]i overload regulated by the binding of ERP44 and IP3R1 is controlled by the pyroptosis mediated by the NLRP3/Caspase-1 pathway, we added Nigericin (an activator of NLRP3) to H/R cells overexpressing ERP44 or silencing IP3R1. Activation of NLRP3 reversed the protective effect of overexpression of ERP44 or inhibition of IP3R1 on H/R-induced cells. The NLRP3/Caspase-1 axis is a typical pathway of cell pyroptosis^44^. A study has reported that the inhibition of pyroptosis can mitigate cerebral ischemia-induced brain injury^45^. These results further confirmed that the increase of [Ca^2+^]i level regulated by ERP44 and IP3R1 can cause H/R-induced cardiomyocyte pyroptosis through the NLRP3/Caspase-1 pathway, thus regulating MI/R injury.

All in all, the current study supported that ERP44 could bind to IP3R1 and inhibit Ca^2+^ overload, thus alleviating pyroptosis, inflammation, and MI/R injury. These results discovered a novel therapy for patients with MI/R. In the future, we shall carry out more prospective trials to refine our clinical guidance for the treatment of MI/R.

## Methods

### Ethics statement

The study got the approval of the Clinical Ethical Committee of Affiliated Hospital of Guangdong Medical University. Informed consent was signed by each eligible participant. All experimental procedures were implemented on the Ethical Guidelines for the Study of Experimental Pain in Conscious Animals.

### Establishment of MI/R and rat grouping

Sprague-Dawley (SD) rats (180–200 g) (Beijing Vital River Laboratory Animal Technology Co., Ltd, SYXK (Beijing) 2017-0033) were anesthetized using 2% pentobarbital sodium (50 mg/kg intraperitoneal) and ventilated with rodent respirators (HX-101E, Taimeng, Chengdu, China) at 80 °C in volume control mode, once a minute. The left axillary was exposed by left thoracotomy at the edge of the left sternum between the 4th intercostal space. The left anterior descending (LAD) coronary artery was ligated about 2 mm from its origin using 6-0 surgical sutures. Occlusion was confirmed by observing the pale myocardium in the left ventricle below the suture line. The rats in the sham group were anesthetized, and the suture was not occluded under the LAD. The rats in the I/R group were ligated with LAD for 30 min and then reperfused for 2 h.

All 84 SD rats were numbered according to the weight and were randomly assigned by random number method to sham group, I/R group, sh-NC (short hairpin RNA-negative control) group, and sh-IP3R1 group, with 21 rats in each group. Three days before the I/R surgery, rats in the sh-NC group were subjected to an intracardiac injection of 30 μL p-LV-sh-NC (Thermo Fisher, Waltham, MA, USA), with the virus titer at 2 × 10^7^ TU/mL, while rats in the sh-IP3R1 group were subjected to an intracardiac injection of 30 μL p-LV-sh-IP3R1 (Thermo Fisher), with the virus titer at 2 × 10^7^ TU/mL. All rats were euthanized 48 h after operation (intraperitoneal injection of 800 mg/kg pentobarbital) and the cardiac tissues were rapidly removed. The cardiac tissues of three rats in each group were used for area at risk (AAR) measurement, 6 for heamatoxylin and eosin (HE) staining and immunohistochemical staining, 6 for Ca^2+^ determination and 6 for other indicators. Each SD rat was selected for any experiment by the researchers who did not participate in the study.

### Measurement of infarct size and AAR

AAR/LV (left ventricle) reflects the degree of myocardial ischemia, while infarct size (INF)/AAR reflects the level of dead myocardium. After the I/R surgery, the suture under the LAD was removed. To identify AAR, rats were perfused with 2 mL 2% Evans blue (Sigma-Aldrich, Merck KGaA, Darmstadt, Germany) through the inferior vena cava. The hearts were removed, washed with phosphate-buffered saline (PBS) solution and frozen at −20 °C. Then, the heart was sliced into five sections at 1 mm, incubated at 37 °C for 15 min with 1% 2,3,5-triphenyltetrazolium chloride (TTC) and PBS solution, and fixed for 2 h with 10% formalin. Image J software analyzed the imaging and calculated the percentage of INF to AAR using a weight-based method.

### Determination of myocardial enzymes

Blood samples were collected from the inner eye angle vein of rats. Serum components were stored at −80 °C for subsequent analysis. Myocardial enzymes creatine kinase isoenzyme MB (CK-MB) and lactate dehydrogenase (LDH) were determined using a semi-automatic biochemical analyzer (Shanghai Biochemical Analysis Instrument Co., Ltd., Shanghai, China).

### HE staining

The left ventricle of heart tissue was fixed in 10% formaldehyde, dehydrated with increasing concentrations of ethanol, embedded in paraffin, and cut into sections at 4 μm. The sections were stained with HE (Solarbio, Beijing, China) after dewaxing, and observed under the light microscope (Leica, Solms, Germany).

### Cardiomyocyte isolation and Ca^2+^ determination

According to a previous study^46^, the cardiomyocytes were isolated by enzyme digestion and loaded with Fluo 4-AM (5 μmol/L; MedChemExpress LLC, NJ, USA) at 37 °C for 20 min. Fluorescence changes of Fluo 4-AM were recorded by confocal microscopy at 25 °C.

In order to measure mitochondrial Ca^2+^, Rhod2 AM (2 μmol/L; MedChemExpress) was cultured in a phenol-free Dulbecco’s modified Eagle’s medium (DMEM) containing 0.03% pluronic acid at 37 °C for 40 min. Then, the cells were washed to remove Rhod2 and degreased in phenol-free DMEM containing 10% FBS. Fluorescence signals were recorded by 561 nm excitation wavelength and 588 emission wavelength under a confocal microscope.

### Immunohistochemistry

The heart sections were dewaxed and hydrated. After antigen repair, the sections were immersed in 3% hydrogen peroxide for 10 min to block the endogenous peroxidase, and blocked with 5% bovine serum albumin (BSA), and incubated with the primary antibody GSDMD (1:1000, ab219800, Abcam, Cambridge, MA, USA) at 4 °C overnight. After that, the sections were incubated with the secondary antibody IgG (1:1000, ab150077, Abcam) for 1 h, and stained with hematoxylin after 2,4-diaminobutyric acid staining. After dehydration and clearing, the sections were observed.

### Reverse transcription-quantitative polymerase chain reaction (RT-qPCR)

The total RNA content from cells was extracted using the one-step method of TRIzol kit (Invitrogen, Carlsbad, CA, USA). High-quality RNA was confirmed by UV analysis and formaldehyde deformation electrophoresis. According to the qRT-PCR kit (ThermoFisher), fluorescent qPCR was performed. PCR primers (Table 1) were designed and synthesized by Sangon Biotech (Shanghai) Co., Ltd. (Shanghai, China). Amplification curve and dissolution curve were confirmed after reaction. The relative expression of miR and mRNAs was calculated by 2^−ΔΔCt^ method, with GAPDH as the internal reference.

**Table 1:** Primer sequences of RT-qPCR.

Gene	Primer sequence
IP3R1	F: 5′-ATGTCTGACAAAATGTCTAG-3′
	R: 5′-GCTTAGGCTGGCTGCTGTGG-3′
ERP44	F: 5′-ATGAATCCTACCGTCTTCCTG-3′
	R: 5′-TTACAGCTCATCTCGATCCC-3′
GAPDH	F: 5′-GGGAGCCAAAAGGGTCAT-3′
	R: 5′-GAGTCCTTCCACGATACCAA-3′

### Western blotting

The total protein content from myocardial tissue or cells was extracted for measurement of protein concentration using the bicinchoninic acid assay (ThermoFisher). The extracted protein was added into the loading buffer and boiled at 95 °C for 10 min. Next, the protein sample (30 μg) was separated by 10% (w/v) electrophpresis and transferred onto polyvinylidene difluoride membranes. The membranes were blocked using 5% BSA for 1 h and cultured with the primary antibodies at 4 °C overnight. After being washed by tris buffered saline tween (TBST), the membranes were incubated with secondary antibody for 1 h and then washed by TBST before enhanced chemiluminescence developing and visualization using Bio-Rad GelEZ imager (Bio-Rad, Inc., Hercules, CA, USA). Image J software (National Institutes of Health, Bethesda, Maryland, USA) was utilized to analyze the gray value of target bands. The antibodies used were IP3R1 (1:2000, ab264281, Abcam), IgG (1:2000, ab205718, Abcam), NLRP3 (1:1000, ab263899, Abcam), ASC (1:1000, ab175449, Abcam), GSDMD (1:2000, ab219800, Abcam), GAPDH (1:1000, ab8245, Abcam), and ERP44 (1:1000, #3798, Cell Signaling Technology, Danvers, MA, USA).

### Detection of Caspase-1 activity

According to the manufacturer’s instructions of the Caspase-1/ICE colormetric assay kit (K111-100, R&D Systems Inc., Minneapolis, MN, USA), the activity of Caspase-1 in rat heart tissues or cells was detected.

### H9C2 cell treatment and grouping

H9C2 cells (ATCC, Manassas, Virginia, USA) were cultured in DMEM supplemented with 1% penicillin, streptomycin, and 10% FBS at 37 °C and 5% CO_2_. When the cell confluence reached above 90%, the cells were passaged. The cells were detached using trypsin, resuspended, and adjusted to 1 × 10^4^ cells/cm^2^, and then incubated at 37 °C in the incubator containing 5% CO_2_. Cells at 3–6 generations were used for the experiment.

si-IP3R1 and si-NC were synthesized by GeneChem (Shanghai, China). ERP44 cDNA was cloned into pcDNA3.1 (Invitrogen) and transfected with Lipofectamine 2000 (Invitrogen) according to the instructions. Nifedipine D6 and Nigericin were purchased from MedChemExpress, and the cells were treated.

H9C2 cells were allocated to blank group (normal H9C2 cells or cardiomyocytes), H/R group (H/R-challenged H9C2 cells or cardiomyocytes), H/R + si-IP3R1 group, H/R + si-NC group, H/R + NC group, H/R + ERP44 group (H9C2 cells or cardiomyocytes transfected with si-IP3R1 or pcDNA3.1-ERP44 were treated with H/R after transfection, with si-NC/pcDNA3.1 as the control), H/R + Nif group (H/R-treated cells were added with 50 μM Nifedipine D6), H/R + si-IP3R1 + Nig group, and H/R + ERP44 + Nig group (10 μmol/L nigericin was added into cells in the H/R + si-IP3R1 or H/R + ERP44 groups, respectively).

### Establishment of H/R cell model and analysis of mitochondrial Ca^2+^ concentration

H9C2 cells were maintained in serum-free DMEM for 2 h and treated with ischemia buffer [118 mM NaCl, 24 mM NaHCO_3_, 1 mM NaH_2_PO_4_ H_2_O, 2.5 mM CaCl_2_ 2H_2_O, 0.5 mM sodium EDTA 2H_2_O, 20 mM sodium lactate, 16 mM KCl (pH = 6.2)]. After being pre-inflated with 95% N_2_ and 5% CO_2_ for at least 5 min, ischemia buffer was added to the cells, and then the cells were placed in a sealed chamber containing deoxygenation reagent. The AnaeroPack system (Mitsubishi Gas Chemical Co. Inc, Tokyo, Japan) was used to produce near-anaerobic conditions. The cells were incubated at 37 °C for 1 h in the incubator containing <1% O_2_ and ~5% CO_2_. Then cells were exposed to near-aerobic conditions for 2 h and incubated under normal conditions (reperfusion) for 24 h. The control cells were cultured in complete medium without ischemia and H/R. The same method was used for isolated adult cardiomyocytes, but the hypoxia time was reduced to 45 min.

Before hypoxia, H9C2 cells and adult cardiomyocytes were loaded with Rhod2. Fluorescence was observed under a microscope. Cell density was determined by crystal violet staining to standardize the intensity.

### Co-immunoprecipitation (Co-IP) assay

Co-IP of IP3R1 and ERP44 was performed as per the instructions of the kit (Thermo Fisher). The cells were dissolved in IP buffer, and the supernatant was incubated with protein A/G agarose beads (Fast Flow, Shanghai Beyotime Biotechnology Co. Ltd., Shanghai, China) at 4 °C for 3 h with anti-IP3R1 antibody or anti-ERP44 with IgG antibody as a control. The agarose beads were washed with cold PBS and the binding protein was eluted. The precipitated protein was determined by western blot analysis.

### Cell count kit-8 (CCK-8)

Cell viability was evaluated by CCK-8 assay (C0038, Beyotime). Cardiomyocytes or H9C2 cells were seeded into 96-well plates at 5000 cells/well. After proper treatment, the medium was replaced with 100 μL CCK-8 solution (containing 90 μL serum-free DMEM and 10 μL CCK-8 reagent). The absorbance at 450 nm of each well was measured.

### Enzyme-linked immunosorbent assay (ELISA)

The levels of IL-1β and IL-18 in tissue or cells were detected according to Rat IL-1β/IL-1F2 Quantikine ELISA kit (RLB00, R&D Systems) and Rat IL-18 ELISA kit (DY521-05, R&D Systems).

### Statistical analysis

Data analysis was introduced using the SPSS 21.0 (IBM Corp., Armonk, NY, USA). Kolmogorov–Smirnov test was employed to assess whether the data were in normal distribution. Data are expressed as mean ± standard deviation. One-way analysis of variance (ANOVA) or two-way ANOVA were applied for the comparisons among multiple groups, and Tukey’s multiple comparison test was applied for the post hoc test after ANOVA. The *p* value was obtained from a two-tailed test, where a value of *p* < 0.05 was indicative of a statistically significance.

## Data Availability

Data supporting present findings are available from the corresponding author upon reasonable request.
